# Factors associated with increased levels of brain natriuretic peptide and cardiac troponin I during the peripartum period

**DOI:** 10.1371/journal.pone.0211982

**Published:** 2019-02-07

**Authors:** Yuki Kimura, Takao Kato, Hiromi Miyata, Issei Sasaki, Eri Minamino-Muta, Yoshinori Nagasawa, Shigeharu Numao, Tadayoshi Nagano, Toshihiro Higuchi, Moriaki Inoko

**Affiliations:** 1 Cardiovascular Center, Tazuke Kofukai Medical Research Institute, Kitano Hospital, Ogimachi, Kita-ku, Osaka, Japan; 2 Department of Cardiovascular Medicine, Kyoto University Graduate School of Medicine, Sakyo-ku, Kyoto, Japan; 3 Department of Obstetrics and Gynecology, Tazuke Kofukai Medical Research Institute, Kitano Hospital, Ogimachi, Kita-ku, Osaka, Japan; 4 Department of Health and Sports Sciences, Kyoto Pharmaceutical University, Kyoto, Japan; Indiana University, UNITED STATES

## Abstract

**Background:**

We aimed to investigate the values and the changes of brain natriuretic peptide (BNP) and cardiac troponin in pregnant women.

**Methods and results:**

We prospectively collected the data of 405 pregnant women who were treated at Japanese general hospital between 2012 and 2013. We analyzed their laboratory data and echocardiographic findings during the third trimester (28–30 weeks’ gestation) and within 4 days of delivery. In addition, we evaluated the factors associated with elevation of BNP and cardiac troponin I (cTnI) levels. The pregnant women were 33.8 ± 5.0 years old and the prevalence of pregnancy induced hypertension (PIH) and placental abnormality was 4.2% and 2.5%, respectively. BNP levels increased after delivery (13.2 pg/mL vs. 23.5 pg/mL; *P* <0.001), correlated with increased left ventricular diastolic dimension (*P* = 0.035), left atrial dimension (*P* <0.001), and decreased hemoglobin (*P* <0.001). Moreover, cTnI levels increased to over 0.015 ng/mL after delivery in 4.0% of pregnant women. In multivariate analysis, PIH (OR: 18.71, *P* = 0.003), placental abnormality (OR: 26.78, *P* = 0.007), and decreased hemoglobin after delivery (OR: 2.59, *P* <0.001) were the factors associated with elevated cTnI.

**Conclusions:**

BNP levels increased in association with cardiac chamber enlargement and decreased hemoglobin after delivery. Additionally, the factors affecting elevated cTnI levels were related to labor and delivery.

## Introduction

Cardiovascular function and hemodynamics dramatically change during pregnancy. The major change related to pregnancy is increase in cardiac output (CO) [1]. This is associated with the rise in heart rate, increase in blood volume, and reduction of systematic vascular resistance during gestation [1–4]. Additionally, reversible cardiac remodeling also occurs, including chamber enlargement [left ventricular diastolic dimension (LVDd) and left atrial dimension (LAD)], annular dilation of valves, and increased LV mass [1, 5, 6]. These changes begin early in pregnancy and reach their peak during second and third trimester (14–27 weeks and 28 weeks–delivery, respectively) [7]. These maternal adaptations are necessary for development of the fetus and protection of the mother from the risks of delivery [1].

In rare instances, pathologic changes occur such as peripartum cardiomyopathy (PPCM) during end of pregnancy or after delivery. PPCM is an idiopathic cardiomyopathy presenting with heart failure secondary to LV systolic dysfunction towards the end of pregnancy or in the months following delivery [8]. Elevated brain natriuretic peptide (BNP) and cardiac troponin levels can help to suspect PPCM [8, 9]. However, the data on concentrations of BNP and cardiac troponin in normal pregnant women is very limited [10]. Therefore, we performed this study to elucidate the sequential changes in BNP levels and cardiac troponin I (cTnI) levels during pregnancy and after delivery.

## Materials and methods

### Participants

We prospectively enrolled 536 pregnant women during the third trimester (28–30 weeks of gestation) at Tazuke Kofukai Medical Research Institute, Kitano Hospital between February 2012 and February 2013. Blood tests and echocardiography were performed during the third trimester and within 4 days of delivery. Women with underlying heart disease (n = 1) and those who did not undergo examinations during the third trimester (n = 38) or within 4 days of delivery (n = 92) were excluded. Finally, we analyzed 405 pregnant women.

### Data collection

The following participant and peripartum characteristics were evaluated: age, history of gravidity or parity, comorbidities [pregnancy induced hypertension (PIH), placental abnormality, and threatened premature delivery], duration of pregnancy and delivery, fetus status, delivery method, amount of bleeding, and maternal morbidity. PIH was defined as systolic blood pressure ≥140 mmHg or diastolic blood pressure ≥90 mmHg. Placental abnormality composed of placental abruption and placenta previa. Threatened premature delivery was suspected in the presence of progressive shortening of cervical length or increased uterine contractions before 37 week of gestation. Additionally, all comorbidities were diagnosed with judgement by expert gynecologists. Amount of bleeding was assessed by obstetrician, gynecologist, and registered nurses. Laboratory data (red blood cell count, hematocrit, hemoglobin, platelet count, BNP, cTnI, and prolactin) and echocardiographic findings [left ventricular ejection fraction (LVEF), LVDd, left ventricular systolic dimension, interventricular septal thickness (IVST), posterior wall thickness, LAD, left atrial volume index (LAVI), inferior vena cava, peak early filling velocity (E), velocity at atrial contraction (A), mitral E/A ratio, deceleration time, velocity of mitral annulus early diastolic motion (e’), and E/e’ ratio] were analyzed during the third trimester and within 4 days of delivery. All examinations were performed by two cardiac sonographers with experience in research echocardiography (S.I. and T.I.) using a standardized protocol [11]. The LVEF was measured using the Teichholz method. Additionally, the factors associated with elevation of BNP and cTnI levels were identified. BNP and cTnI levels were measured using the chemiluminescent enzyme immunoassay (Abbott Corp.). The normal level of BNP was ≤18.4 pg/mL in this assay. Elevated cTnI was defined as >0.015 ng/mL (normal level, ≤0.015 ng/mL, which contains the 99%tile). All relevant data are within the manuscript.

### Ethics

Written informed consent was obtained from all participants. The research protocol was approved by the institutional review board of Kitano Hospital according to the ethical guidelines of the 1975 Declaration of Helsinki and its amendments (P12-12-002). The patient records/information was anonymized prior to analysis.

### Statistics

Categorical variables were expressed as numbers and percentages. Continuous variables were expressed as means ± standard deviations or medians and interquartile range (IQR). When comparing between prepartum and postpartum data, the paired t-test was used for continuous variables. The correlation between the changes in laboratory and echocardiographic data was carried out using the Pearson’s product-moment. Univariate and multivariate analyses were performed to investigate the factors associated with elevated cTnI, and the odds ratios (ORs) and 95% confidence intervals (CIs) were computed using the logistic regression model. A *P*-value <0.05 was considered statistically significant in all analyses. Statistical analyses were performed using JMP software, version 10 (SAS Corp., Cary, NC).

## Results

### Participants’ and peripartum characteristics

Baseline data of the 405 pregnant women are presented in Table 1. The mean age was 33.8 ± 5.0 years and advanced maternal age (≥35 years) was observed in 47%. The prevalence of PIH and placental abnormality (placental abruption or placenta previa) was 4.2% and 2.5%, respectively. Regarding the fetus status, the mean duration of pregnancy was 38.7 ± 2.7 weeks and the incidence of twin gestation was 3.5%. Caesarean section accounted for 16% of deliveries and approximately one third of women underwent induction of labor using oxytocin (119/125; 95%), dinoprost (10/125; 8%) or balloon catheterization (38/125; 30%). The median blood loss during delivery was 392 mL (IQR: 250–612 mL). There were no symptom of heart failure and maternal morbidity in all women during pregnancy or within 4 days of delivery.

**Table 1:** Prenatal and peripartum characteristics.

	All subjects (n = 405)
Prenatal characteristics	
Age (year)	33.8 ± 5.0
Advanced maternal age^a^ [n (%)]	190 (47)
Gravidity (times)	1.42 ± 0.71
Parity (times)	1.41 ± 0.70
Primipara [n (%)]	258 (64)
Comorbidities	
PIH [n (%)]	17 (4.2)
Placental abnormality [n (%)]	10 (2.5)
Threatened premature delivery [n (%)]	12 (3.0)
Fetus status	
Duration of pregnancy (week)	38.7 ± 2.7
Birth weight (g)	2981 ± 475
Twin gestation [n (%)]	14 (3.5)
FGR [n (%)]	8 (2.0)
NRFS [n (%)]	5 (1.2)
Delivery	
Caesarean delivery [n (%)]	65 (16)
Induction of labor [n (%)]	125 (31)
Oxytocin	119 (24)
Dinoprost	10 (2.5)
Balloon catheter	38 (9.4)
Blood loss during delivery[median (IQR), mL]	392 (250–612)
Duration of delivery [median (IQR), hours]	7.3 (4.3–12.2)
Maternal morbidity [n (%)]	0 (0)

^a^Advanced maternal age defined as maternal age ≥35 years.

PIH, pregnancy induced hypertension; FGR, fetal growth restriction; NRFS, non-reassuring fetal status; IQR, interquartile range.

### Laboratory data and echocardiographic findings before and after delivery

**Fig 1:**
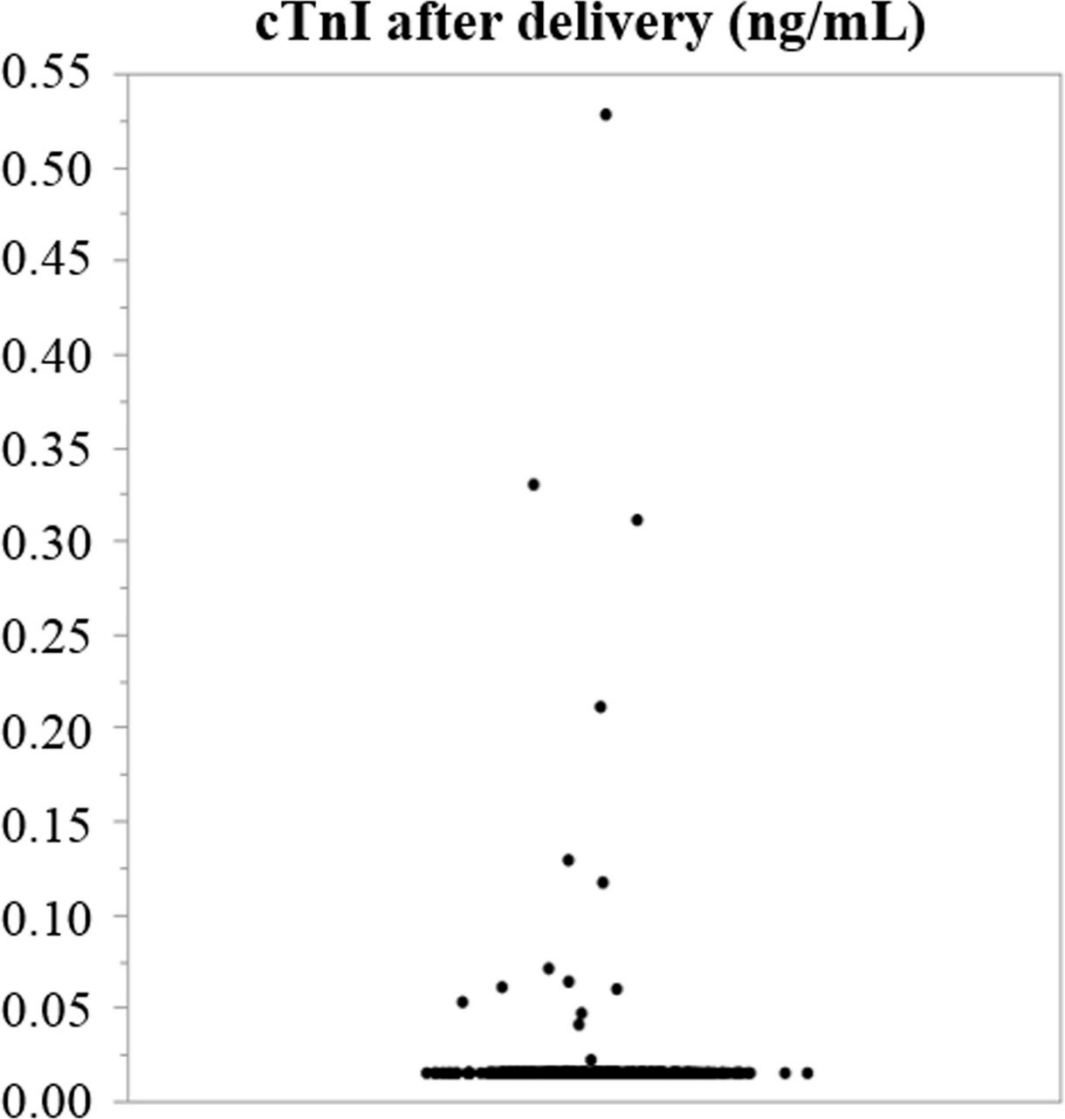
The distribution of cardiac troponin I levels after delivery. cTnI, cardiac troponin I.

The results of examinations (blood tests and echocardiography) during the third trimester and within 4 days of delivery are summarized in Table 2. The mean hemoglobin levels had decreased after delivery (111.7 ± 10.1 g/L vs. 100.5 ± 14.3 g/L; *P* <0.001). There was a positive correlation between the decrease of hemoglobin and blood loss during delivery (P <0.001, R = 0.27). All pregnant women had normal cTnI levels during the third trimester, but cTnI levels had increased to over 0.015 ng/mL after delivery in 4.0% (Fig 1). Additionally, BNP levels also increased after delivery (13.2 pg/mL, IQR: 8.5–20.8 pg/mL vs. 23.5 pg/mL, IQR: 14.0–38.2; *P* <0.001), and went over the normal level (18.4 pg/mL) in 226 women (56%).

**Table 2:** Results of examinations during pregnancy and after delivery.

	During pregnancy (n = 405)	After delivery (n = 405)	Amount of change	*P* value
Laboratory data				
RBC (10^12/L)	3.63 ± 0.31	3.50 ± 0.49	-0.098 ± 0.57	<0.001
HCT (L/L)	0.33 ± 0.027	0.30 ± 0.041	-0.025 ± 0.052	<0.001
Hb (g/L)	111.7 ± 10.1	100.5 ± 14.3	-10.8 ± 15.5	<0.001
Plt (10^9/L)	231.9 ± 51.1	242.7 ± 60.7	13.1 ± 49.4	<0.001
BNP [median (IQR), pg/mL]	13.2 (8.5–20.8)	23.5 (14.0–38.2)	9.6 (-0.6–22.2)	<0.001
cTnI (ng/mL)	0.015	0.015 (0.015–0.015)	0 (0–0)	0.01
Elevated cTnI^a^ [n (%)]	0 (0)	16 (4)		
PRL [median (IQR), ng/mL]	196.6 (136.6–261)	281 (208.7–346.3)	76.3 (16.3–148.4)	<0.001
Echocardiographic findings				
LVEF (%)	62.8 ± 3.6	62.9 ± 3.3	0.10 ± 4.27	0.32
LVDd (mm)	47.1 ± 3.5	48.4 ± 3.5	1.31 ± 2.76	<0.001
LVDs (mm)	31.2 ± 2.6	31.9 ± 2.5	0.98 ± 4.01	<0.001
IVST (mm)	6.51 ± 0.91	6.75 ± 0.10	0.25 ± 1.28	<0.001
PWT (mm)	6.65 ± 0.88	6.97 ± 0.91	0.34 ± 1.25	<0.001
LAD (mm)	31.9 ± 3.5	32.6 ± 3.9	0.73 ± 3.54	<0.001
LAVI (mL/m^2^)	17.1 ± 3.9	19.0 ± 4.5	0.69 ± 9.53	<0.001
IVC (mm)	11.2 ± 3.2	12.4 ± 3.1	0.84 ± 7.09	<0.001
E/A ratio [median (IQR)]	1.50 (1.26–1.83)	1.40 (1.21–1.70)	-0.10 (-0.40–0.20)	<0.001
E DT [median (IQR), ms]	187.0 (163.0–208.0)	179.5 (157.3–201.0)	-5.50 (-34.0–21.5)	<0.001
e’ (cm/s)	12.5 ± 2.4	11.5 ± 2.4	-1.06 ± 3.21	<0.001
E/e’ ratio [median (IQR)]	6.4 (5.7–8.0)	7.3 (6.1–8.9)	0.7 (-0.4–2.0)	<0.001

RBC, red blood cell; HCT, hematocrit; Hb, hemoglobin; Plt, platelet; BNP, brain natriuretic peptide; cTnI, cardiac troponin I; PRL, prolactin; LVEF, left ventricular ejection fraction; LVDd, left ventricular diastolic dimension; LVDs, left ventricular systolic dimension; IVST, interventricular septal thickness; PWT, posterior wall thickness; LAD, left atrial dimension; LAVI, left atrial volume index; IVC, inferior vena cava; E, peak early filling velocity; A, velocity at atrial contraction; DT, deceleration time; e’, velocity of mitral annulus early diastolic motion.

^a^Elevated cTnI was defined as >0.015 ng/mL.

On echocardiography, no pregnant women had a reduced LVEF (<50%) and no significant change in LVEF occured during the peripartum period. However, cardiac chamber size and wall thickness significantly enlarged after delivery (LVDd: 47.1 ± 3.5 mm vs. 48.4 ± 3.5 mm; *P* <0.001; IVST: 6.51 ± 0.91 mm vs. 6.75 ± 0.10 mm; *P* <0.001; and LAVI: 17.1 ± 3.9 ml/m^2^ vs. 19.0 ± 4.5 ml/m^2^; *P* <0.001). LV diastolic function was slightly reduced after delivery (e’: 12.5 ± 2.4 cm/s vs. 11.5 ± 2.4 cm/s; *P* <0.001).

### Factors associated with elevation of BNP and cTnI levels

**Fig 2:**
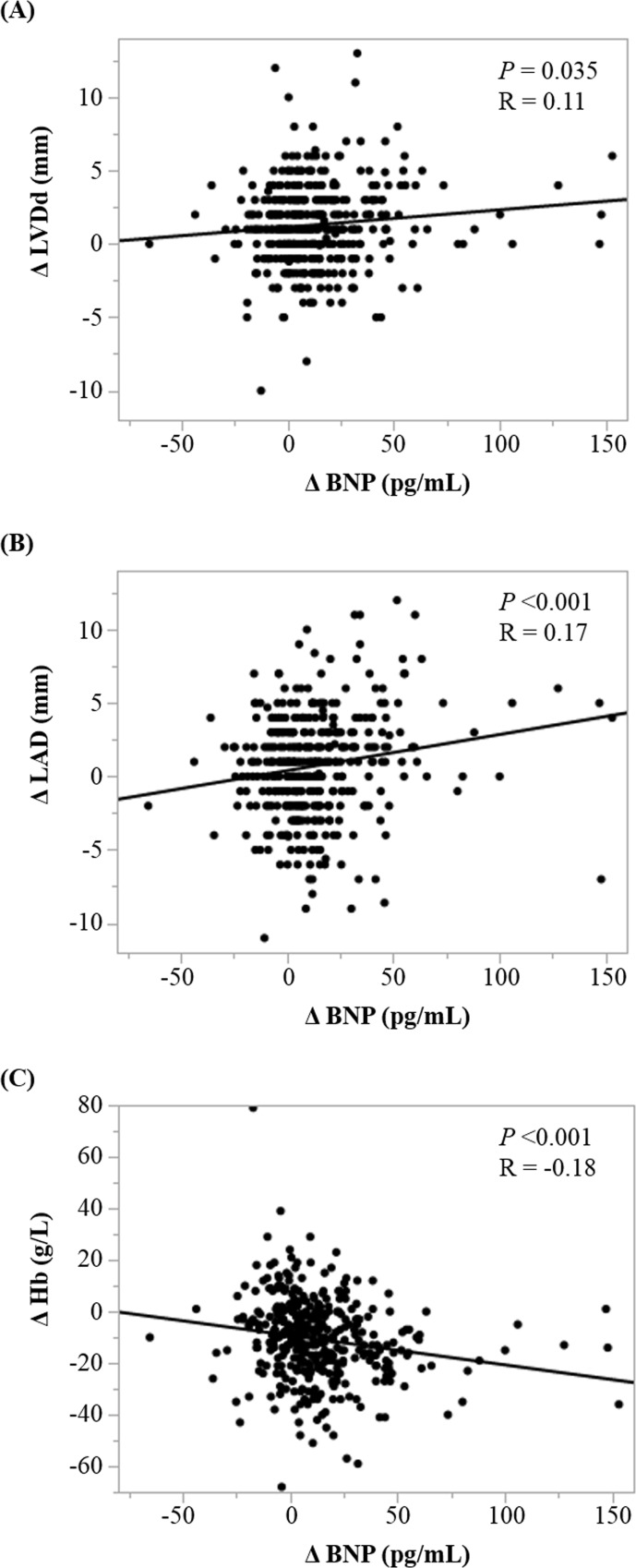
Factors correlated with increase of brain natriuretic peptide levels. (A) Correlation between Δ LVDd and Δ BNP (*P* = 0.035, R = 0.11). (B) Correlation between Δ LAD and Δ BNP (*P* <0.001, R = 0.17). (C) Correlation between Δ Hb and Δ BNP (*P* <0.001, R = 0.18). Δ represents change from the third trimester to postpartum period. BNP, brain natriuretic peptide; LVDd, left ventricular diastolic dimension; LAD, left atrial dimension; Hb, hemoglobin.

The increase in BNP levels was positively correlated with an increase in LVDd (*P* = 0.035) and LAD (*P* <0.001), and negatively correlated with an increase in hemoglobin (*P* <0.001) (Fig 2). However, the increase in BNP levels did not have significant correlations to an increase in TnI levels (p = 0.62) and prolactin (P = 0.056). No other statistically significant correlations were observed.

In univariate analyses, the factors associated with elevated cTnI were age (OR: 1.14 per 1 year, 95% CI: 1.02–1.28, *P* = 0.016), PIH (OR: 6.16, 95% CI: 1.58–24.12, *P* = 0.009), induction of labor (OR: 3.69, 95% CI: 1.06–12.86, *P* = 0.04), blood loss (OR: 1.002 per 1 mL, 95% CI: 1.001–1.003, *P* <0.001), and decreased hemoglobin after delivery (OR: 1.97 per hemoglobin -1 g/L, 95% CI: 1.41–2.74, *P* <0.001). In multivariate analysis, PIH (OR: 18.71, 95% CI: 2.63–133.06, *P* = 0.003), placental abnormality (OR: 26.78, 95% CI: 2.51–285.69, *P* = 0.007), and decreased hemoglobin after delivery (OR: 2.59 per hemoglobin -1 g/L, 95% CI: 1.38–4.85, *P* <0.001) were the independent factors affecting elevated cTnI (Table 3).

**Table 3:** Factors associated with elevated cardiac troponin I levels after delivery.

Factors	Univariate		Multivariate	
	OR (95%CI)	*P* value	OR (95%CI)	*P* value
Age (per 1 year)	1.14 (1.02–1.28)	0.016	1.02 (0.85–1.21)	0.84
Advanced maternal age^a^	1.62 (0.55–4.79)	0.38		
PIH	6.16 (1.58–24.12)	0.009	18.71 (2.63–133.06)	0.003
Twin gestation	4.48 (0.91–21.93)	0.065	1.97 (0.20–19.08)	0.56
Placental abnormality	8.56 (0.84–87.16)	0.07	26.78 (2.51–285.69)	0.007
Induction of labor	3.69 (1.06–12.86)	0.04	1.65 (0.36–7.65)	0.52
Blood loss during delivery (per 1 mL)	1.002 (1.001–1.003)	<0.001	1.000 (0.999–1.002)	0.45
Decreased Hb after delivery (per Hb -1 g/L)	1.97 (1.41–2.74)	<0.001	2.59 (1.38–4.85)	<0.001
BNP after delivery (per 1 pg/mL)	1.005 (0.986–1.023)	0.64		
PRL after delivery (per 1 ng/L)	1.001 (0.999–1.003)	0.44		
LVEF after delivery (per 1%)	1.006 (0.865–1.169)	0.94		
LVDd after delivery (per 1 mm)	1.02 (0.89–1.18)	0.74		
LAD after delivery (per 1 mm)	1.08 (0.95–1.23)	0.25		
E/A ratio after delivery (per 1)	0.83 (0.20–3.46)	0.80		
e’ after delivery (per 1 cm/s)	0.83 (0.68–1.02)	0.082	0.94 (0.68–1.30)	0.71

^a^Advanced maternal age defined as maternal age ≥ 35 years.

OR, odds ratio; CI, confidence interval; PIH, pregnancy induced hypertension; Hb, hemoglobin; BNP, brain natriuretic peptide; PRL, prolactin; LVEF, left ventricular ejection fraction; LVDd, left ventricular diastolic dimension; LAD, left atrial dimension; E, peak early filling velocity; A, velocity at atrial contraction; e’, velocity of mitral annulus early diastolic motion.

## Discussion

The main findings of the present study are as follows: 1) A small proportion of peripartum women showed elevated cTnI levels, which was associated with factors related to induction of labor and delivery; 2) BNP levels increased in association with dilation of cardiac chamber size and decreased hemoglobin after delivery.

Normal pregnancy and delivery are accompanied with multiple physiological changes and adaptations in almost every organ system to aid in fetal growth and delivery. For instance, the major hematologic change observed is physiological anemia. Indeed, red blood cell counts, and hematocrit and hemoglobin levels in the present study were decreased compared to the normal range. During pregnancy, both red blood cell mass and plasma volume increase to meet the metabolic demands of the uterus, prevent reduced CO due to impaired venous return in the supine or standing position, and prepare for the bleeding during delivery [12, 13]. However, dilutional anemia occurs because plasma volume increases to a greater extent than red blood cell mass (30–50% vs. 20–30%) [12–14]. In the present study, hemoglobin level decreased after delivery because of bleeding, on the ground that the decrease in hemoglobin levels correlated with the amount of blood loss during delivery. It takes 6 weeks for physiologic anemia of pregnancy and delivery to resolve [15].

In the present study, no pregnant women had LV systolic dysfunction during the peripartum period. This is because the incidence of PPCM in Japan is lower than that in the USA (1/20000 births vs. 1/3000–4000 births) [16]. Similarly, other indicators of cardiac function were normal despite physiological changes. As demonstrated by previous reports [5], the changes in cardiac structure, such as chamber enlargement, valve annular dilation, and increased LV mass, are reversed as early as 2 weeks after delivery [5]. We did not observe the reversion of these changes back to normal since the echocardiographic assessment was conducted within only 4 days of delivery.

The release of BNP increases in patients with heart failure since ventricular stretching due to increased filling pressure stimulates BNP secretion [17]. During a normal pregnancy, BNP levels are approximately 2-fold higher than those in non-pregnant status and do not significantly fluctuate during pregnancy and after delivery (4–6 weeks postpartum) [10]. However, the present study demonstrates that BNP levels increase by approximately 1.8-fold immediately after delivery. This increase in BNP levels was associated with chamber enlargement and progress of decreased hemoglobin. These findings indicate that LV stretching caused by plasma volume increase and heart strain due to bleeding during delivery increase BNP levels. However, this change in BNP levels is significantly lower than that observed in patients with heart failure, such as those with PPCM [8, 10, 18]. Therefore, BNP levels during pregnancy and after delivery should be useful as an indicator of PPCM.

Serum cardiac troponin is a specific and sensitive biomarker of cardiac injury that can detect even minor myocardial injury [19]. Cardiac troponin can neither cross the placenta, due to its molecular size, nor can it be produced in the placenta [20, 21]. Therefore, the elevated cTnI after delivery could indicate myocardial injury during delivery. In the present study, PIH, placental abnormality, and decreased hemoglobin after delivery were the factors associated with elevated cTnI levels. PIH is a hypertensive disorder during pregnancy closely linked to preeclampsia. In pregnant women with preeclampsia, placental hypoperfusion caused by abnormalities in the development of the placental vasculature induces the release of antiangiogenic factors, which impair vascular endothelial function in the placental and maternal circulation [22–24]. Moreover, increased sensitivity to angiotensin II has been reported in preeclampsia [22, 23]. These changes could lead to myocardial injury due to microcirculatory disorders in addition to hypertension’s effects. Indeed, previous articles reported elevated cTnI levels and increased risk of cardiovascular disease during postpartum follow-up in pregnant woman with PIH [20, 25]. Additionally, the risk factors for placental abruption are advanced age, multiple gestation, and PIH, which are also risk factors for PPCM [26–29]. Additionally, placenta previa is related to increased bleeding during delivery and the subsequent anemia. In addition to bleeding, which increases the vascular resistance, increased blood pressure during pregnancy might lead to temporal hypoperfusion and subsequent myocardial injury [30, 31]. These factors are closely linked and affect each other. Thus, subclinical myocardial injury might occur during the peripartum period if PIH, placental abnormality, and anemia after delivery are present.

This study has several key limitations that should be discussed. First, there may be variabilities in echocardiographic data between two cardiac sonographers. Second, the pregnant women in this study were older than the general population because our institution is a general hospital. Therefore, selection bias during participant enrollment cannot be excluded. Third, the levels of troponin during pregnancy were below the detection limit in all pregnant women, and some of the women showed the above the limit after delivery. Therefore, we could not calculate the delta (the change from the third trimester to after delivery). On the other hand, the levels of BNP increase, decrease, or did not change after delivery. For that reason, we selected the categorical variable for troponin and the continuous for BNP in the analysis. Finally, the number of pregnant women with elevated cTnI was not enough to perform multivariate analysis. Therefore, this study might be prone to statistical errors, especially in multivariate analysis.

In conclusion, BNP and cTnI levels after delivery were increased compared to those at 28–30 weeks’ gestation. Moreover, the factors affecting elevated cTnI levels were associated with labor and delivery. Additionally, BNP levels increases were related to cardiac chamber enlargement and decreased hemoglobin after delivery. The data about changes in biomarkers during normal pregnancy is important in assessing cardiac accident in pregnant women. Therefore, these findings suggested that elevated cardiac biomarkers in normal pregnant women may be helpful to suspect myocardial overload and damage and subsequent cardiovascular events during peripartum period.
